# Identification of success factors in elite wrestlers—An exploratory study

**DOI:** 10.1371/journal.pone.0247565

**Published:** 2021-03-04

**Authors:** Igor Cieśliński, Dariusz Gierczuk, Jerzy Sadowski

**Affiliations:** Faculty of Physical Education and Health in Biala Podlaska, University of Physical Education, Warsaw, Poland; Universidad de Almería, SPAIN

## Abstract

Identification of success factors in wrestling as well as establishing their hierarchy are crucial from a cognitive and practical standpoint. It may provide a lot of practical recommendations related to wrestling-specific training. The aim of this study was to identify and establish the hierarchy of success factors in wrestling regardless of a fighting style and weight class. This study included 168 elite male freestyle and Greco-Roman wrestlers. They were divided into two groups: athletes who won medals (successful wrestlers) in high-rank competitions (Polish Championships or higher) and those who did not win any medals (less successful wrestlers) in those competitions. The following elements were assessed: anthropological measurements, body composition, dynamic strength, strength endurance, agility, special endurance, wrestling-specific fitness, response time, technical wrestling skills and anaerobic capacity. For initial data analysis, one-way ANOVA (α = 0.005) was used. Random Forests classifier was employed to identify success factors and to determine the importance of each of these factors in terms of sports performance. Seven key success factors were identified: anaerobic power, strength endurance, response time, special endurance, wrestling-specific fitness and technical wrestling skills performed in a horizontal position. Random Forests turned out to be an effective method of modelling success in wrestling (compared to SVM and KNN, which were also used in the study). These findings suggest that wrestling-specific training can be effectively monitored by controlling several vital indicators of athletes’ preparedness: anaerobic power, strength endurance, response time, special endurance, wrestling-specific fitness and technical wrestling skills (the performance of reverse waistlock from a standing position and trunk grip gut wrench assessed by experts).

## Introduction

Wrestling is one of the oldest recorded combat sports and one of the first sports included in the programme of ancient and modern Olympic Games. There are two competitive styles of wrestling–Greco-Roman and freestyle [1]. The main difference is that Greco-Roman wrestlers are forbidden from performing any holds below the waist, whereas in freestyle they are allowed to use their whole body during the fight [2]. A great body of studies dealing with differences in physical, physiological or psychological profiles between wrestling styles revealed that there are no significant differences between Greco-Roman and freestyle wrestling [3–5]. A few studies, however, have found some differences between both wrestling styles. For example, Bayraktar and Koc [6] and Demirkan et. al. [7] found differences for mesomorph values, elastic strength, agility, speed abilities, leg strength and flexibility. Identifying the success factors (physical, physiological and psychological ones) as well as establishing their hierarchy, together with wrestling-specific fitness and other elements contributing to wrestling performance would be beneficial to coaches. Numerous researchers focused on examining differences mainly in single success factors, e.g. anthropometric measurements of wrestlers at various levels of competition [8, 9], development of motor abilities [3, 7, 10], technical and tactical wrestling skills [3, 11, 12] or physical performance [3, 10, 11, 13] in the novice-expert domain. These studies showed that anthropometric measurements [14, 15], upper and lower body strength, anaerobic power, strength endurance [3, 7, 10, 11, 13, 16], response time [17] and technical wrestling skills such as suplex throw, throw over the hip (head and arm) and rear takedown by front waistlock [12, 16, 18] differentiated successful and less successful wrestlers of both styles [12, 16]. Surprisingly, a lot of researchers omitted other important components of sports preparation, e.g. sport-specific skills. It is common knowledge that a lot of factors contribute to success in competition but wrestling-specific fitness and technical wrestling skills are vital in successful wrestlers’ profile.

Moreover, in a few studies that simultaneously analysed anthropometric, physiological and neuromuscular factors as well as wrestling-specific fitness and technical wrestling skills for successful and less successful wrestlers of different weight classes and performance levels, traditional analytical methods were applied [7, 16, 18, 19, 20]. Roemmich and Frappier [19] found that grip strength of the left hand, flexibility of the low back and hamstring, push-ups, strength of the right quadriceps and aerobic endurance (12-min. run) were of importance in achieving wrestling success. In another study that employed a logistic regression analysis, García-Pallarés et al. [16, 18] showed that only two factors, i.e. fat-free mass and one repetition maximum (1 RM) strength were the most important predictors of successful female wrestling performance. In males, they identified such factors as training experience, fat free mass, one repetition maximum (1RM) strength, muscle power in bench press, squat exercise and Wingate test peak power [16, 18]. From a practical point of view, these studies provide ambiguous and only partially valuable results. One possible explanation was that wrestling-specific fitness and technical wrestling skills were not sufficiently emphasised in the analyses. Moreover, standard methods (mainly linear models) were used for statistical analysis. The use of such standard methods is certainly possible; however, it is only justified when their limitations are considered. For instance, if we increase the likelihood of rejecting the null hypothesis from the commonly used value of 0.05 to 0.005 because it reduces the false positive rate [21, 22], it will also show that a great deal of research ought to be reinterpreted in terms of the correctness of conclusions. Such an approach is being questioned more and more often [23]. The use of machine learning methods that do not require meeting the criteria of linearity and distribution normality may help to avoid such limitations and identify success factors more accurately. To date, these methods have rarely been applied in sports sciences [24–27].

Due to the above-mentioned limitations, the purpose of this study was to identify and establish the hierarchy of success factors in wrestling regardless of a fighting style and weight class.

## Material and methods

Elite male freestyle (n = 74) and Greco-Roman (n = 94) wrestlers took part in the study. The wrestlers were 20.42 ± 2.50 years old and the length of their training experience was 7.76 ± 2.60 years. The participants were divided into two groups. The first group consisted of athletes who won medals in Polish Junior and Senior Championships and in two international competitions (successful, n = 85), while the other group included wrestlers who finished 5^th^-8^th^ in those competitions (less successful, n = 83) [28]. Demographic and anthropometric features of the whole research group are shown in Table 1.

**Table 1:** Characteristics of the research group *(mean ± SD*, *Me)*.

	Age (years)		Training experience (years)		Body mass (kg)		Stature (cm)	
	*mean ± SD*	*Me*	*mean ± SD*	*Me*	*mean ± SD*	*Me*	*mean ± SD*	*Me*
**wrestlers (n = 168)**	20.42±2.50	20	7.76 ± 2.60	7	76.53 ± 15.20	74.3	175.87 ± 7.26	175.5
**successful (n = 85)**	20.91 ± 2.93	21	8.67 ± 2.85	8	77.92 ± 16.49	75.8	175.38 ± 7.98	175
**less successful (n = 83)**	19.93 ± 1.87	19	6.83 ± 1.94	6	75.11 ± 13.71	72.2	176.37 ± 6.46	176

All the participants gave their written informed consent to take part in the study. The study was approved by the Ethics Committee of the University of Physical Education in Warsaw (SKE 01-01/2016).

### Procedures

The study involved anthropometric measurements as well as assessment of body composition, dynamic strength, strength endurance, agility, wrestling-specific fitness and special endurance, response time, technical wrestling skills and anaerobic capacity. Reliability of the tests assessing conditioning levels was checked by means of the ‘test-retest’ method. For this purpose, 30 Greco-Roman wrestlers were randomly selected for the study. The subjects were 19.6 ± 1.92 years of age and the length of their training experience was 8.4 ± 2.43 years. The tests were carried out twice, with an interval of 5 days.

The measurement conditions were the same for all competitors. Twenty-four hours before the tests, the wrestlers did not participate in any intensive sports training. The study was conducted for 5 consecutive days: day 1 –anthropometrics and body composition (7.00–11.00), dynamic strength, strength endurance (15.00–18.00), day 2 –response time (9.00–13.00), wrestling-specific fitness (15.00–18.00), day 3 –agility (9.00–13.00), anaerobic capacity–crank arm (15.00–18.00), day 4 –technical wrestling skills (9.00–13.00), anaerobic capacity–crank leg (15.00–18.00), day 5 –special endurance (9.00–13.00 and 15.00–18.00). Prior to the tests, the wrestlers performed a standard warm-up. This warm-up took 20 minutes and was completed 35 minutes before testing. It consisted of 3 x 20 m jogging and skipping with walking back, 3 x 20 m submaximal sprinting, 3 x 20 m sprint from drills; 2 x 20 m leg swings, fast feet and high knees, 3 x 10 m maximal sprints, 30 seconds of mixed calisthenics (press-ups, dead bugs, planks) and 2 minutes of dynamic stretching.

#### Anthropometric measurements and body composition assessment

Anthropometric measurements included standing height and body mass, length of upper and lower limbs, width of shoulders and pelvis, circumferences of arm, forearm, thigh and calf, skinfold thickness (chest, biceps, subscapular, suprailiac, abdominal, calf and chin). The measurements were performed in accordance with guidelines from the International Society for the Advancement of Kineanthropometry [29]. Moreover, BMI (Quetelet II) was calculated. Standing height, length of upper and lower limbs, width of shoulders and pelvis, circumferences of arm, forearm, thigh and calf were measured to the nearest 0.1 cm, body mass to the nearest 0.1 kg and skinfold thickness was assessed using a skinfold caliper (Holtain Ltd., UK), accurate to 0.2 mm [30]. Body composition (the content of fat, muscles and water) was determined by means of bioelectrical impedance (single frequency of 50 kHz, hand held, four electrodes; RJL BIA 101/S Body Impedance Analyzer, Akern, Clinton Township, MI). All the measurements and calculations were made in the morning. The wrestlers had not consumed any foods or beverages prior to the examinations.

#### Dynamic strength

Standing broad jump: the participants stood with their toes behind the take-off line in the starting position. They were instructed to choose their initial squatted position in 45° knee flexion or 90° knee flexion to see if it was altered in relation to the score obtained. By bending the knees with free swinging of the arms one time or with restricted arms, the participants jumped forward to cover the greatest distance possible. The distance was measured from the take-off line to the rearmost heel (cm). Test reliability (ICC) was 0.93.

#### Strength endurance

Pull-ups: the participants placed their hands in a pronated position at a distance of 1.5 times of their bi-acromial width on an overhead pull-up bar and pulled their body up to the bar by adducting at the shoulder joints and flexing at the elbows. When the bottom of the chin reached the level of the bar, they returned to the starting position. Test reliability (ICC) was 0.92.

Wall bar hanging leg raises: the participants hung down a gymnastic wall bar with their back against the wall. When given the signal to start, each participant raised both legs simultaneously so that the knees touched the chest and returned to the initial position. The result was the number of repetitions performed in 30 seconds. Test reliability (ICC) was 0.89.

#### Agility

**Fig 1:**
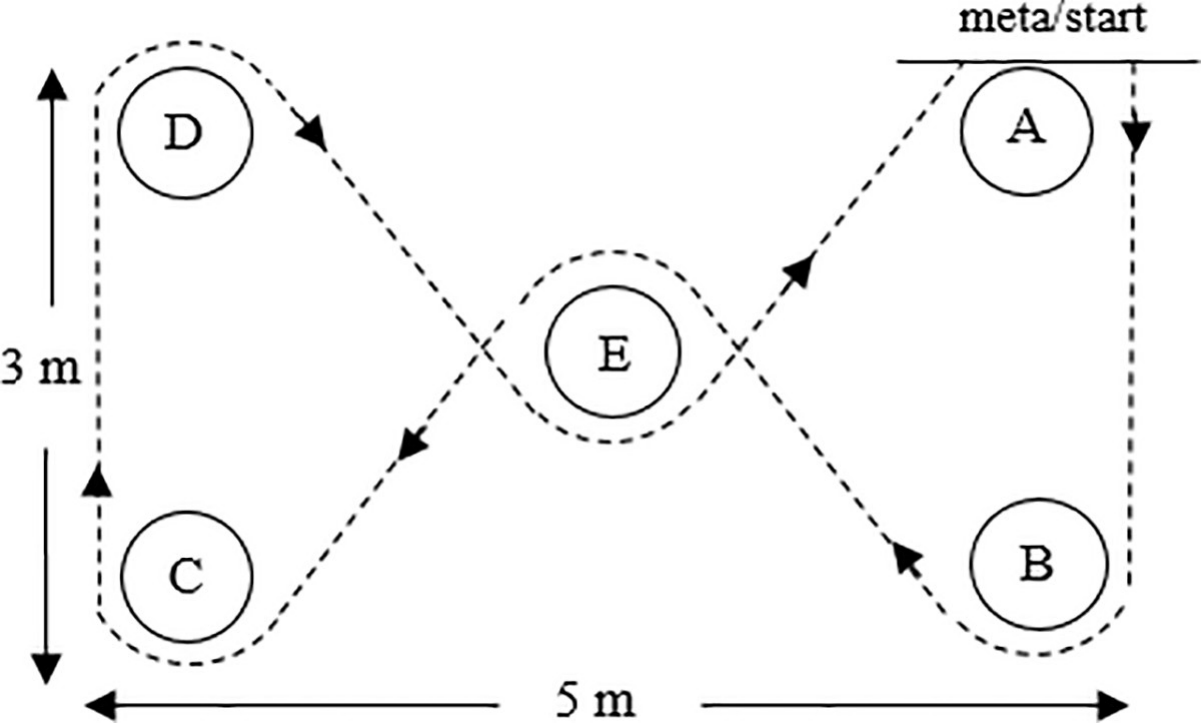
Illustration of the zigzag (‘envelope’) run.

Zigzag (‘envelope’) run: the participants stood in front of flag A. When given the signal to go, each person ran along lines A-B-E-C-D-E-A and around the flags without touching any (Fig 1). When the participant crossed the finish line after performing three courses, the time was stopped. The result was the total time of covering the distance three times consecutively (s). Test reliability (ICC) was 0.77.

#### Wrestling-specific fitness

Bridge circles each athlete assumed a horizontal position with his forehead pressed against the mat and his feet positioned by the line marked on the mat (front bridge). When given the signal to start, the wrestler made several steps to the right, threw over his left leg and took the back bridge position without changing the position of his head. After that, he repeated his steps in the same direction, thew over his right leg and returned to the initial position. Each participant performed the routine three times to the left and then three times to the right. The result was the time needed to perform the whole routine (s). Test reliability (ICC) was 0.74.

Standing gymnastic bridge with return: the wrestler was in a forward stance on the mat; when given the signal to start, he performed backward bend (bridge) and returned to the initial position. He repeated the routine three times. The result was the total time of exercise performance, i.e. from the moment of receiving the signal to go to the moment of returning to the initial position (s). Test reliability (ICC) was 0.63.

#### Special endurance

Suplex throws of the mannequin: standing in the middle of the mat, the wrestler held the mannequin by the waist and, when given the signal to start, he performed suplex throws. The test involved two 3-minute rounds with a 1-minute interval in between. Within each 3-minute round, there were four 45-sec. sections. Each section consisted of a period of 30 s where the mannequin was thrown four times at an easy pace, followed by 15 s of throws performed at a maximum pace. Lightweight wrestlers (50–75 kg) used a 20 kg mannequin, whereas heavyweight competitors (76–125 kg) did the test using a 30 kg mannequin. The result was the sum of throws performed during the two rounds. Test reliability (ICC) was 0.85.

#### Response time

Simple reaction: reaction time and movement time were measured using the RT test (S1 version) from the Vienna Test System (VTS) [31]. Sitting in front of the computer screen, the participant put the index finger of his dominant hand on a sensor called the rest key on the response panel. He was instructed to press the reaction key (a rectangular black button) on seeing a specific stimulus (yellow light) on the screen. The routine was repeated 28 times within 2 minutes. The result was mean RT (time between the onset of the stimulus and the release of the rest key, expressed in ms) and mean MT (time between releasing the rest key and pressing the reaction key, expressed in ms). Test reliability was 0.71 for reaction time and 0.78 for movement time.

Response time was measured using two Batak Lite tests (tests IV and V). Batak Lite is a device with the following dimensions: 1143 x 1800 x 950 mm. The device has eight light signals. Test IV–standing in front of the device, the participant had to press 8 randomly lit buttons as many times as possible in 30 seconds. The score was the number of lights pressed. Test V–standing in front of the device, the participant had to press as many randomly lit lights as possible in 2 minutes. The score was the number of lights pressed. Test reliability (ICC) was 0.58 and 0.68.

#### Technical wrestling skills

To assess technical wrestling skills, three elements performed in a vertical position (throw over the hip (head and arm), throw over the shoulder, rear takedown by front waistlock) and two elements performed in a horizontal position (trunk grip gut wrench and reverse waistlock from a standing position) were employed [32]. A 10-point scale was applied for evaluation. The following aspects were taken into account: the correctness of the initial and final positions, grip technique, movement harmony and movement amplitude (in the case of throws). Penalty points were given for technical errors and subtracted from 10 points. For instance, the penalties were applied for the following errors: incorrect initial and final positions (0.5 pts), incorrect grips (0.5 pts), unnecessary movements while performing a particular skill (0.5–1 pts), low amplitude during a throw (0.5 pts) or no movement harmony (0.5–1 pts). Evaluation was carried out by five experienced wrestling judges. An average of three scores was taken for analysis. Two extreme scores (the highest and the lowest ones) were excluded. The concordance coefficient of judges’ scores was 0.82.

#### Anaerobic capacity

Anaerobic capacity of upper and lower limb muscles was measured with the 30-sec. Wingate test [33]. The test was preceded by a 5-minute warm-up on a cycle ergometer with two 10-sec. acceleration phases (in the 2nd and 3rd minute of the warm-up). The test took place five minutes after the warm-up. The arm cranking test was performed in a sitting position on an 814 Monark ergometer (Sweden) and the leg cycling test was performed on a Monark 874E ergometer (Sweden). Before each test, the crank set was calibrated according to the manufacturer’s recommended procedure. The height of the central axis of the arm ergometer and crank-arm length were adjusted according to the optimal proportions determined previously (crank length 12–12.5% of arm span and crank-axle height between 50 and 60% of the wrestler’s height) [34]. Workload was equal to 55 g/kg (of body mass) for upper limbs and 75 g/kg for lower limbs. The crank arm trials were 30 s in duration and participants were instructed to crank as powerfully as possible on each revolution throughout the trial and not to adopt any pacing strategy. The break between the tests for upper limbs and lower limbs lasted at least 24 hours. In both tests, Peak power (W_peak_), total work (W_total_) and the time of achieving and maintaining peak power of upper and lower limb muscles were registered using the computer programme MCE version 5.1. Peak power was defined as the greatest power value recorded by the devices used in the study, while total work was defined as the total work done during 30 seconds.

### Data analysis

**Fig 2:**
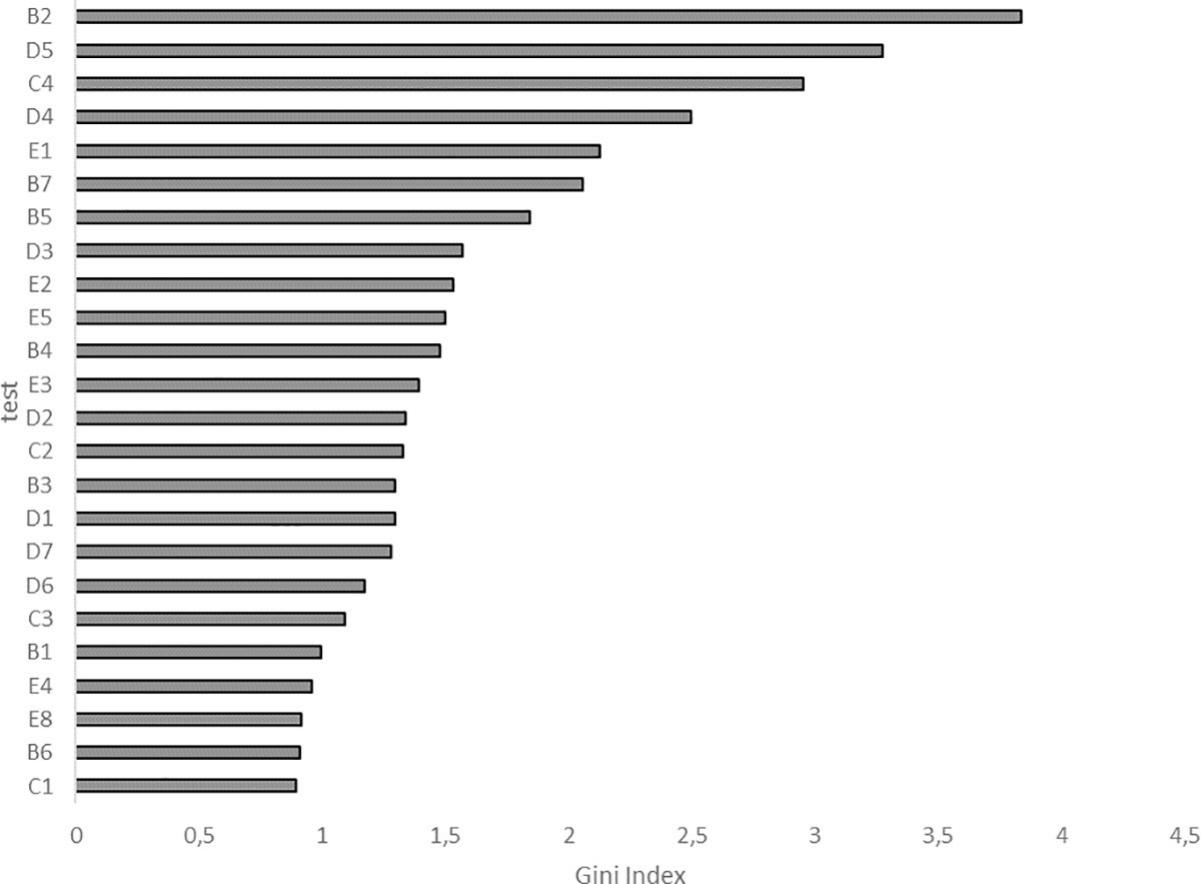
Path of data analysis.

The first basic descriptive statistics were calculated for wrestlers divided into two groups (successful and less successful). Furthermore, one-way ANOVA was carried out when its assumptions of normality and homogeneity of variance were met. Normality and homogeneity were checked using the Shapiro-Wilk test and Bartlett test, respectively. If at least one of the assumptions was violated, the Kruskal-Wallis test was employed. Machine learning (random forest (RF), support vector machine (SVM), k-nearest neighbour (KNN)) was used as the main analysis method. It was verified in terms of a prediction error (classification efficiency) [35, 36]. Variable importance was determined using the Gini Index (GI). RF is an ensemble method based on connecting many decision trees created for randomly selected variable subsets (in various subgroups, the same variables may occur) and linking them in such a way that error is minimised and prediction efficiency is maximised. RF can be used even for very small datasets where the number of variables is larger than the number of rows [37]. Variables were also classified by means of SVM with hyper parameters as well as KNN algorithm (Fig 2). Finally, the obtained models were compared with regard to their classification efficiency, see [35, 36, 38].

The described procedure also aimed to make it possible to construct a model in such a way that it would describe chances of achieving sports success depending on the values of variables referring to anthropometric characteristics and fitness indices. Moreover, an attempt was made at selecting those tests which would provide maximum information that would help to assess chances of achieving the highest sports results.

## Results

In the beginning, the analysis aimed to verify the hypothesis that there are no differences between freestyle and Greco-Roman wrestlers (one-way ANOVA). The hypothesis was rejected only in the case of four variables describing sports technical wrestling skills (throw over the leg, rear takedown by front waistlock, trunk grip gut wrench, reverse waistlock from a standing position). It enabled us to carry out further analysis of success factors together for both groups. Results (one-way ANOVA again obtained by both groups (successful, less successful)) in particular tests are shown in Table 2. It was noted that the greatest effect (ω^2^/ eta^2^) occurred in the following indices: D4 –trunk grip gut wrench (0.183), D5 –reverse waistlock from a standing position (0.161), B2 –pull ups (0.142), B7 –suplex throws of the mannequin (0.15), B4 –wall bar hanging leg raises (0.14).

**Table 2:** Differences between successful and less successful wrestlers in tests performed, groups’ descriptive statistics and effect size.

Test / index	Successful (mean ± SE)	less successful (mean ± SE)	aoa	p	ω^2^/eta^2^
**A1 –Body height (cm)**	175.3±0.86	176.37±0.71	T		0.001
**A2 –Body mass (kg)**	77.9±1.79	75.1±1.51	F		0.005
**A3 –BMI (kg/m**^**2**^**)**	25.1±0.40	24.1±0.39	F		0.02
**A4 –Length of lower limbs (cm)**	98.2±0.64	98.5±0.61	F		0.006
**A5 –Length of upper limbs (cm)**	71.6±0.52	73.3±0.60	T		0.018
**A6 –Shoulder width (cm)**	44.2±0.039	45.7±0.41	T		0.032
**A7 –Pelvis width (cm)**	34.6±0.29	34.8±0.28	T		0.004
**A8 –Arm circumference (cm)**	32.1±0.39	30.5±0.36	F		0.04
**A9 –Forearm circumference (cm)**	27.1±0.41	26.1±0.33	F		0.015
**A10 –Thigh circumference (cm)**	56.9±0.74	54.8±0.51	F		0.018
**A11 –Calf circumference (cm)**	35.6±0.43	36.2±0.34	F		0.001
**A12 –Sum of 7 skinfolds (cm)**	56.8±2.30	62.8±2.70	F		0.011
**A13 –Fat content (%)**	9.75±0.37	8.67±0.44	F		0.03
**A14 –Muscle mass (kg)**	66.4±1.21	64.1±0.99	F		0.000
**A15 –Water content (%)**	62.9±0.35	64.8±0.39	T	*	0.075
**B1 –Standing broad jump (cm)**	251.5±2.03	244.9±2.06	T		0.024
**B2 –Pull-ups (n)**	33.7±0.99	26.5±0.91	T	*	0.142
**B3 –Zigzag (‘envelope’) run (s)**	20.3±0.13	20.5±0.14	T		0.002
**B4 –Wall bar hanging leg raises (n)**	32.2±0.36	29.7±0.35	F	*	0.14
**B5 –Bridge circles (s)**	12.2±0.24	13.2±0.26	F		0.051
**B6 –Standing gymnastic bridge with return (s)**	6.8±0.21	7.5±0.18	F	*	0.048
**B7 –Suplex throws of the mannequin (n)**	49.8±0.53	46.1±0.46	F	*	0.15
**C1 –Simple reaction (reaction time) (ms)**	234.8±2.95	236.2±2.31	F		0.00
**C2 –Simple reaction (movement time) (ms)**	95.7±1.78	102.0±1.56	T		0.048
**C3 –Response time—test IV (n)**	49.0±0.55	47.1±0.54	T		0.028
**C4 –Response time–test V (n)**	188.0±1.23	181.6±1.15	F	*	0.091
**D1 –Throw over the hip (head and arm) (pts)**	7.1±0.09	6.5±0.12	F	*	0.05
**D2 –Throw over the shoulder (pts)**	6.9±0.11	6.2±0.11	F	*	0.10
**D3 –Rear takedown by front waistlock (pts)**	6.9±0.12	6.3±0.11	F	*	0.096
**D4 –Trunk grip gut wrench (pts)**	6.6±0.10	5.7±0.09	T	*	0.183
**D5 –Reverse waistlock from a standing position (pts)**	6.9±0.11	6.1±0.09	T	*	0.161
**E1 –Peak power of upper limb muscles (Watt/kg)**	8.5±0.08	8.1±0.07	T	*	0.085
**E2 –Total work of upper limb muscles (J/kg)**	203.7±1.55	196.1±1.62	T	*	0.058
**E3 –Time of achieving peak power of upper limb muscles (s)**	7.2±0.21	7.2±0.19	F		0.00
**E4 –Time of maintaining peak power of upper limb muscles (s)**	5.0±0.16	5.0±0.14	F		0.00
**E5 –Peak power of lower limb muscles (Watt/kg)**	11.1±0.17	10.2±0.17	F	*	0.07
**E6 –Total work of lower limb muscles (J/kg)**	251.6±3.12	238.8±3.43	F	*	0.049
**E7 –Time of achieving peak power of lower limb muscles (s)**	5.6±0.16	5.9±0.21	F		0.00
**E8 –Time of maintaining peak power of lower limb muscles (s)**	3.4±0.13	3.6±0.16	F		0.00

Key: ω^2^/eta2 –omega/kw eta squared effect size

*—p < 0.005

aoa–if the assumptions of anova have been met

The correctness of assigning wrestlers to groups on the basis of the analysed variables (Table 2) (after finishing the learning process) proved to be highly diverse depending on the method applied (Table 3). The best results were obtained using Random Forests and Support Vector Machine with Gaussian kernel (18–23% of incorrect classifications); however, two cases (two wrestlers) randomly selected from each group (not used in the process of learning or testing) were properly classified by Random Forests algorithm, while SVM incorrectly classified one of the non-medallists as medallists (thus, its prediction efficiency came to 50% only). Therefore, the results of the analysis were presented using RF.

**Table 3:** Results of variable classifications vs. algorithms used.

Method	Mean classification error (%)	Parameters for the best model
Random Forests	18.75	Ntree = 300; nvstart = 0.5nv
SVM		
no tuning	28.88	
gauss	23.12	gamma = 0.5; cost = 1
polynomial	28.24	degree = 2, cost = 1, gamma = 0.5
sigmoid	43.07	gamma = 1, cost = 0.1
linear	28.02	gamma = 0.03, cost = 0.1
KNN	26.96	k = 5

**Fig 3:**
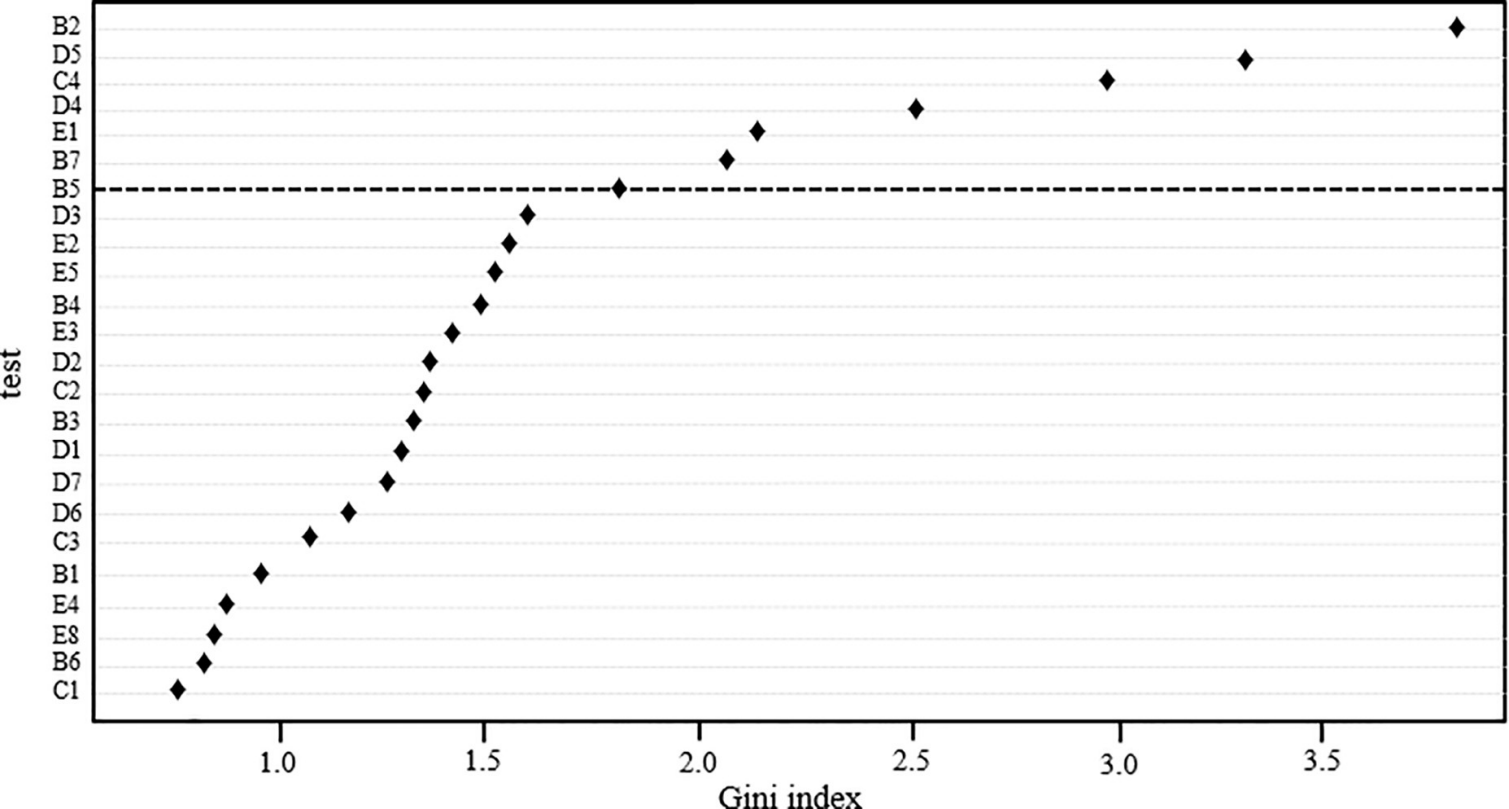
Importance of particular variables in differentiating the groups of successful and less successful wrestlers.

The following variables mainly determine the classification of wrestlers depending on sports success (in the sense of a reduction in mean squared error–GI): strength endurance (B2), technical wrestling skills (D5), response time (C4), technical wrestling skills (D4), peak power of upper limb muscles (E1), special endurance (B7), wrestling-specific fitness (B5) (Fig 3).

Repeated performance of the Random Forests analysis for only 7 out of 39 identified variables (indices B2, D5, C4, D4, E1, B7 and B5) that best classify successful and less successful athletes provided additional information within the revealed variables. The classification error totalled 21.43%, which means it was greater only by approx. 4%, when the number of variables fell (from 39 to 7) by as much as 83.4%. In this case, the classification produced better results for successful (20.0% of the classification error) than less successful wrestlers (23.0% of the classification error). Moreover, the order of importance of the 7 variables changed. The final hierarchy was as follows: E1 (GI = 5.52), B2 (GI = 5. 42), C4 (GI = 4.20), B7 (GI = 3.31), B5 (GI = 3.03), D5 (GI = 2.94) and D4 (GI = 2.88).

## Discussion

The aim of this study was to identify and establish the hierarchy of success factors in wrestling regardless of a fighting style and weight class. Seven success factors were identified. The most important factors for wrestling performance were peak power of upper limb muscles (E1), strength endurance (B2), response time (C4), special endurance (B7), wrestling-specific fitness (B5) and technical wrestling skills performed in a horizontal position (D5 and D4). As expected, the values of the above-mentioned factors were higher in successful wrestlers than in less successful ones.

Peak power of upper limb muscles (E1) ranked top in the hierarchy of success factors. Previous studies reported that anaerobic power is a crucial variable for achieving high-level wrestling performance and accurately discriminates between successful and less successful wrestlers regardless of their age, weight category and wrestling styles. Numerous researchers agree that without high levels of anaerobic power, wrestlers cannot execute technical manoeuvres or control opponents’ offensive actions [5, 13, 39–42]. García-Pallarés et al. [18] reported that out of the 30 examined variables, only three were logical (logistic regression was used) including peak power. It is in line with the findings of our study. Nikooie et al. [10] revealed that upper-limb mean power was higher in successful elite male wrestlers compared with their less successful counterparts [10].

Strength endurance (B2) proved to be the next factor in the hierarchy of success factors. Several studies reported that high strength endurance is the key factor leading to performance success in wrestling. Nikooie et al. [10] noted that successful junior wrestlers performed approximately 29% more pull-ups than less successful ones. The same observation was recorded in senior athletes, with higher sit-up and pull-up test results in successful wrestlers compared with less successful ones. If we consider that different indications of strength (including relative strength) determine conditioning preparation of wrestlers, the pull-up test is its good measure, which is in line with the results obtained by other researchers [43].

A small number of researchers [17, 44–46] examined response time closely related to wrestling performance. The views regarding the role of response time in a wrestling fight are ambiguous [17, 46, 47]. Gierczuk et. al. [17], Whitley and Montano [46] claimed it is a success factor in wrestling. In contrast to them, Kroll [47] did not find any differences in response time between successful and less successful athletes. In our study, response time to the light signal (C4) was identified as an important success factor in wrestling. It is logical because during a fight, wrestlers have to register, process and counteract appropriately to different actions of opponents. Therefore, the time to respond is of crucial importance in wrestling and was identified in the cluster of success factors. Whitley, Montano [46], Gierczuk, Bujak [44], Gierczuk et al. [17] share the view that wrestlers’ performance depends on response time. Although the role of response time in wrestling may seem obvious, there is still not enough information about it. Surprisingly, a number of researchers neglected it in their investigations [7, 10, 16, 18].

Special endurance (B7) also constituted a part of the model created by success factors. This factor was assessed with suplex throws of the mannequin that was performed during two 3-minute rounds, similar to the duration of an official wrestling fight. During competition, wrestlers have to maintain high intensity of competitive actions for an extended period of time [10, 43]. Such intensive manoeuvres require high strength-endurance levels [5, 39, 43]. Special endurance (in the form of throws that resemble conditions of a wrestling fight) is considered to be a key element in conditioning preparation in wrestling. It exerts a positive influence on the effectiveness of a fight and speed of energetic recovery, which may lead to higher performance [40, 41].

It was also interesting to observe that successful wrestlers had significantly higher results than less successful athletes in another important wrestling skill, i.e. bridge circles (B5). It is considered to be a wrestling-specific skill for both wrestling styles and it is often regarded as a canon of training in wrestlers at various levels of competition. Its performance requires strong cervical muscles, which are actively involved in wrestling [48, 49]. Essentially, wrestlers need well-developed cervical extensor muscles to counter the opponent’s offensive and defensive actions and to maintain the neck and the head in a fixed position against the contestant’s force [49]. For that reason, many researchers consider improving cervical muscles performance a substantial value to wrestlers [48, 49]. Furthermore, massive cervical muscles extension force is needed by the wrestler when tackled to avoid touching the mat with their shoulders. Overall, it can be claimed that isometric strength is one of the key factors leading to high-level performance success in wrestling, which was confirmed in our research.

In agreement with previous investigations, our research revealed that technical wrestling skills, i.e. reverse waistlock from a standing position (D5) and trunk grip gut wrench (D4) were also indicated as important success factors [3, 12, 16, 50, 51]. This is mainly attributed to the fact that higher muscle mass and anaerobic power can help the wrestler perform technical manoeuvres more easily.

From the perspective of monitoring training process effectively, it is enough to use seven variables that made up the model of success factors in wrestling. The comparisons of tests that were similar in terms of error reduction e.g. indices E1 and B2 (peak power of upper limb muscles and pull-ups), C4 and B7 (response time and suplex throws of the mannequin) as well as B5 and D5 (bridge circles and reverse trunk hip throw) and D5 and D4 (reverse trunk hip throw and trunk grip trunk grip gut wrench) underpin our suggestion. It may act as another guidance mechanism to reduce the revealed factors to just a few, which may translate into minimising the costs (both indirect and direct ones) of monitoring the level of the wrestler’s performance. The above-mentioned results generally corroborate findings from previous studies. However, a unique value of the current study is that the hierarchy of success factors for wrestlers in both styles was highlighted. A different order of success factors compared to the findings of other researchers [7, 16, 18, 19, 20] may stem from the fact that success factors common for both styles were revealed in our study. Moreover, a different method of analysis (Random Forests) was applied. It is worth noting that the linear model (ANOVA) and the ML algorithm (Random Forests–the most effective algorithm in our study) provided the basis for a similar interpretation of the study results. Both approaches revealed the central importance of such indices as B2, D4 and D5. However, in both methods, the importance of particular indices is attached slightly differently. In the case of RF, indices E1 and C4 were very important. They were also significant in ANOVA, yet here their effect size was rather low (0.09 and 0.08, respectively). In turn, B4 had high effect size in ANOVA (0.14) and medium importance in RF. Despite these slight discrepancies, it can be noted that the most essential indices are the ones that refer to basic conditioning abilities, selected technical skills and anaerobic capacity. It is notable that the factors also predict the probability of an athlete being successful in wrestling competitions, regardless of the wrestling style and weight category.

There are some limitations to this study that are worth noting. Firstly, the wrestlers from our study were not Olympic medallists, but athletes successful on a national level only. Secondly, only male wrestlers were investigated. Future investigations should be carried out in the context of gender and the competitive level of wrestlers and their achievements in international sports competitions.

## Supporting information

S1 File Raw data set. (CSV)
